# Plasma protein thiolation index (PTI) as a potential biomarker for left ventricular hypertrophy in humans

**DOI:** 10.1371/journal.pone.0216359

**Published:** 2019-05-08

**Authors:** Begoña Quintana-Villamandos, Irene González del Pozo, Laia Pazó-Sayós, Jose María Bellón, Álvaro Pedraz-Prieto, Ángel G. Pinto, Emilio Delgado-Baeza

**Affiliations:** 1 Departamento de Anestesiología, Hospital General Universitario Gregorio Marañón, Madrid, Spain; 2 Departamento de Farmacología y Toxicología, Facultad Medicina, Universidad Complutense de Madrid, Madrid, Spain; 3 Departamento de Estadística, Instituto de Investigación Sanitaria Gregorio Marañón, Madrid, Spain; 4 Departamento de Cirugía Cardiovascular, Hospital General Universitario Gregorio Marañón, Madrid, Spain; 5 Unidad de Medicina y Cirugía Experimental, Instituto de Investigación Sanitaria Gregorio Marañón, Madrid, Spain; International University of Health and Welfare, School of Medicine, JAPAN

## Abstract

**Background:**

Left ventricular hypertrophy (LVH) has been associated with oxidative stress, although not with the protein thiolation index (PTI). This study explored the potential use of PTI as a biomarker of oxidative stress in patients with LVH.

**Methods:**

We recruited 70 consecutive patients (n = 35 LVH and n = 35 non-LVH) based on an echocardiography study in our institution (left ventricular mass indexed to body surface area). Plasma levels of both S-thiolated protein and total thiols were measured as biomarkers of oxidative stress by spectrophotometry, and PTI was calculated as the molar ratio between S-thiolated proteins and the total thiol concentration.

**Results:**

Values for plasma S-thiolated proteins were higher in patients with LVH than in the control group (P = 0.01). There were no differences in total thiols between the LVH group and the control group. Finally, PTI was higher in patients with LVH than in the control group (P = 0.001). The area under the ROC curve was 0.75 (95% CI, 0.63–0.86; P<0.001), sensitivity was 70.6%, and specificity was 68.6%, thus suggesting that PTI could be used to screen for LVH. A multivariable logistic regression model showed a positive association (P = 0.02) between PTI and LVH (OR = 1.24 [95% CI, 1.03–1.49]) independently of gender (OR = 3.39 [95% CI, 0.60–18.91]), age (OR = 1.03 [95% CI, 0.96–1.10]), smoking (OR = 5.15 [95% CI, 0.51–51.44]), glucose (OR = 0.99 [95% CI, 0.97–1.01]), systolic arterial pressure (OR = 1.10 [CI 1.03–1.17]), diastolic arterial pressure (OR = 0.94 [CI 0.87–1.02]), dyslipidemia (OR = 1.46 [95% CI, 0.25–8.55]), estimated glomerular filtration rate (OR = 0.98 [95% CI, 0.96–1.01]), body mass index (OR = 1.03 [95% CI, 0.90–1.10]), and valvular and/or coronary disease (OR = 5.27 [95% CI, 1.02–27.21]).

**Conclusions:**

The present study suggests that PTI could be a new biomarker of oxidative stress in patients with LVH.

## Introduction

Left ventricular hypertrophy (LVH) is a mechanism by which the heart adapts to different types of stress [1]. LVH progresses over time, thus increasing the incidence of heart failure, malignant arrhythmias, myocardial ischemia, systolic dysfunction, sudden death, and cerebrovascular complications [2–5]. Certain pharmacological treatments produce regression of LVH and decrease the incidence of cardiovascular adverse events and mortality in affected patients [6–11]. Therefore, early diagnosis and initiation of appropriate treatment are essential.

Electrocardiography (ECG) and echocardiography are the most widely used procedures for the diagnosis of LVH, although ECG shows low sensitivity and echocardiography takes longer, is more expensive, and is not always available for technical reasons [12]. 21 studies show that the accuracy of electrocardiographic indexes in the diagnosis of LVH is unsatisfactory [13]. The ECG shows an area under the ROC curve of 0.55 with 14.7% sensitivity and 96.7% specificity [14] and the echocardiography shows an area under the ROC curve of 0.62 with 90% sensitivity and 25% specificity [15]. In clinical practice, echocardiography is the imaging technique of choice [16]. However, the cost-effectiveness of routine echocardiography for diagnosis of LVH has been debated [17].

Biomarkers of oxidative stress play a relevant role in diagnosis, evaluation of disease status, and assessment of the health-enhancing effects of therapies. The protein thiolation index (PTI) is a new biomarker of oxidative stress that is defined as the molar ratio of S-thiolated proteins to free protein thiols in plasma [18]. The PTI can be determined easily and inexpensively and could be used for routine for therapeutic monitoring in clinical practice [18]. LVH has been associated with oxidative stress [19], although not with PTI. The hypertrophic remodeling of the myocardium incorporates increased cardiomyocyte growth, reactive interstitial fibrosis and enhanced nitro-oxidative stress. Oxidative stress occurs when excess reactive oxygen species (ROS) are generated that cannot be adequately countered by intrinsic antioxidant systems. ROS are derived from many sources including mitochondria, xanthine oxidase, uncoupled nitric oxide synthases and NADPH oxidases. Growing evidence implicates redox-sensitive pathways in the development of LVH [20]. LVH shows intensified formation of ROS that include decreased NO (nitric oxide) production due to the oxidation of the endothelial NO synthase, reduced NO bioavailability and oxidation of the sGC enzyme (soluble guanylate cyclase) rendering it a hem-free and NO-insensitive form [21]. The protective role of the guanylate cyclase (GC)–cyclic guanosine; monophosphate (cGMP)- protein kinase G (PKG) pathway in the cardiovascular system has been investigated [22]. The pharmacological activation of the sGC—cGMP pathway may exert reverse-remodeling properties in the myocardium and the intensified formation of ROS interferes with the above mentioned pathway [23].

This study explored the potential use of PTI as a biomarker of oxidative stress in patients with LVH.

## Materials and methods

### Study participants

We performed a observational study of 70 patients referred consecutively to our institution (to cardiac surgery section) in the period of time December 2015 to March 2016. When a patient is referred to cardiac surgery section, an echocardiography study is usually performed. The first patient, if the echocardiographic study showed LVH, this patient was included in the LVH group and if the echocardiographic study did not show LVH, the patient was included in the control group, and thus with the second patient referred to this section, and then with the third and so on, and then we have done the blood test to study PTI. In the period of time December 2015 to March 2016, 81 patients were referred to the cardiac surgery section, but we included 70 (11 patients show exclusion criteria. The clinical research ethics committee of the Hospital General Universitario Gregorio Marañon approved the study, and all participants gave their written informed consent to undergo the procedures. The inclusion criterion was age ≥18 years. The exclusion criteria were hemodialysis, rheumatoid arthritis, and alkaptonuria. A clinical history was taken, an echocardiography study was performed, and values for S-thiolated protein and total thiols were determined. The echocardiography was performed using the iE33 system (Philips, CA, USA) equipped with a S5-1 probe (1–5 MHz). The echocardiographic diagnosis of LVH (based on left ventricular mass indexed to body surface area, LVMbs) was consistent with the recommendations of the American Society of Echocardiography [24] and was used to form the two study groups (LVH and non-LVH, 35 patients each).

### Measurement of PTI in clinical samples

#### Blood collections and plasma preparation

Blood samples (2.4 mL) were drawn from the antecubital vein and collected in Vacutainer tubes (BD, Plymouth, UK) containing citrate (300 𝜇L) before being centrifuged at 900*g* for 10 minutes at 4°C to obtain plasma, which was aliquoted and stored at –80°C for further analysis.

#### Quantification of PTI

Plasma levels of both S-thiolated protein and total thiols were measured individually as biomarkers of oxidative stress by spectrophotometry in the same sample. Total thiols were assessed using the microplate 5,5´-dithiobis (2-nitrobenzoic acid) (DTNB) assay [25], and absorbance was measured at 412 nm in a Synergy HT Multi-Mode Microplate Reader. Plasma S-thiolated proteins were determined by spectrophotometry using a new method with Ninhydrin reagent, as previously described [18]. PTI was calculated as the molar ratio of S-thiolated proteins to the concentration of free, DTNB-titratable SH group proteins (total thiols) [18].

### Statistical methods

Categorical variables were expressed as frequency and percentage, and groups were compared using the Pearson chi-square test or Fisher´s test as required. Normality was assessed using the Kolmogorov–Smirnov test. Quantitative variables were described as mean ± standard deviation (for continuous, normally distributed variables), and the between-group comparison was made with two-sample t-test and the Mann-Whitney U-test as appropiate. A receiver operating characteristic curve (ROC) was constructed to determine the diagnostic reliability of PTI. A cut-off point was chosen for the diagnosis of LVH, and their sensitivity, specificity, predictive values, and likelihood ratios were calculated with their respective confidence intervals. A univariate and multivariable logistic regression model was performed to study the strength of the association between PTI and LVH. The independent variables were gender, age, smoking, glucose, systolic and diastolic arterial pressure, estimated glomerular filtration rate (eGFR), body mass index (BMI), coronary disease and valve disease. P values <0.05 were considered statistically significant. The analysis was performed using IBM SPSS Statistics for Windows, Version 21.0 (IBM Corp, Armonk, NY, USA) and Prism Graph Pad 6.0 (Graph Pad Software, California, USA).

## Results

In total, 70 patients were included (35 diagnosed with LVH by echocardiography). The patients were considered to have systemic hypertension if they has a history of elevated blood pressure requiring long-term therapy, they were considered to have dyslipidemia if they had the diagnosis of dyslipidemia requiring long-term treatment and they were considered to have diabetes if they had the diagnosis of diabetes requiring long-term treatment. The clinical characteristics of patients are shown in Table 1. No significant differences were observed between the groups for demographic and cardiovascular risk factors (gender, age, weight, body mass index, smoking, diabetes mellitus, hypertension, and dyslipidemia), organ disease (renal failure, coronary disease, valvular disease, coronary and valvular disease, and systolic dysfunction), or antihypertensive treatment (angiotensin receptor blockers, angiotensin-converting enzyme inhibitors, β-blockers, calcium channel blockers, and diuretics). As reported in Table 1, there were significant differences in left ventricular mass index between the groups (LVH and no LVH, 141.8± 45.6 and 88.7±25.1 g/m^2^ respectively, P<0.001).

**Table 1:** Clinical charecteristics of the study patients.

	LVH (n = 35)	No LVH (n = 35)	*P*	OR (95% CI)
Age (years)	69.7 (10.2)	63.5 (14.7)	0.06	1.04 (1–1.09)
Gender, M (%)	23 (65.7)	21 (60)	0.509	0.72 (0.27–1.92)
Weight (kg)	76.1 (13.8)	75.4 (14.9)	0.623	1.01 (0.98–1.04)
Body mass index (m^2^)	1.8 (0.1)	1.8 (0.2)	0.824	0.43 (0.04–5.03)
LVMI (g/m^2^)	141.8 (45.6)	88.7 (25.1)	<0.001	1.05 (1.02–1.07)
Proteins (g/dL)	6.9 (0.7)	6.9 (0.6)	0.841	0.92 (0.43–1.99)
Glucose (mg/dL)	115.3 (38)	109.8 (36.9)	0.667	1.00 (0.99–1.02)
Hemoglobin (g/dL)	12.9 (2.1)	13.4 (2)	0.333	0.89 (0.70–1.13)
Hematocrit (%)	38.6 (5.8)	39.8 (5.6)	0.375	0.96 (0.88–1.05)
Platelets x10^3^ /μL	211 (67)	196 (59)	0.304	1 (1–1.01)
ALT (U/L)	22.2 (19.9)	19.1 (10.6)	0.432	1.02 (0.99–1.04)
GGT (U/L)	28.5 (20.1)	29.7 (20.5)	0.805	1.00 (0.99–1.01)
Total bilirubin (mg/dL)	0.69 (0.5)	0.73 (0.5)	0.771	0.88 (0.36–2.13)
CK (U/L)	67.2 (56.4)	62.4 (38.4)	0.849	1.00 (0.99–1.01)
Smokers (%)	4 (11.4)	6 (17.1)	0.734	1.55 (0.4–6.07)
Diabetes mellitus (%)	11 (31.4)	7 (20)	0.243	0.52 (0.18–1.57)
Hypertension (%)	28 (80)	23 (65.7)	0.116	0.41 (0.13–1.27)
Dyslipidemia (%)	22 (62.8)	17 (48.6)	0.176	0.52 (0.20–1.35)
Renal failure (%)	9 (25.7)	10 (28.5)	0.611	1.11 (0.39–3.2)
Coronary disease (%)	1 (2.8)	3 (8.5)	0.310	4.20 (0.40–44.4)
Vavular disease (%)	22 (62.8)	15 (42.8)	0.155	0.17 (0.02–1.65)
Coronary and valvular (%)	12 (34.2)	17 (48.5)	0.264	1.73 (0.66–4.55)
Systolic Disfunction (%)	13 (37.1)	14 (40)	0.881	1.08 (0.41–2.83)
Antihypertensive (%)				
ARB	7 (20)	3 (8.5)	0.188	0.36 (0.09–1.54)
ACEi	14 (40)	13 (37.1)	0.731	0.84 (0.32–2.22)
β-Blockers	12 (34.2)	11 (31.4)	0.733	0.84 (0.31–2.29)
Calcium channel blockers	11 (31.4)	6 (17.1)	0.143	0.43 (0.14–1.35)
Diuretics	15 (42.8)	10 (28.5)	0.179	0.51 (0.19–1.38)

LVH, left ventricular hypertrophy; LVMI, left ventricular mass index. ALT, alanine transaminase. GGT, gamma-glutamyl transpeptidase. CK, creatine kinase. ARB, angiotensin receptor blockers; ACEi, angiotensin-converting enzyme inhibitors.

**Fig 1:**
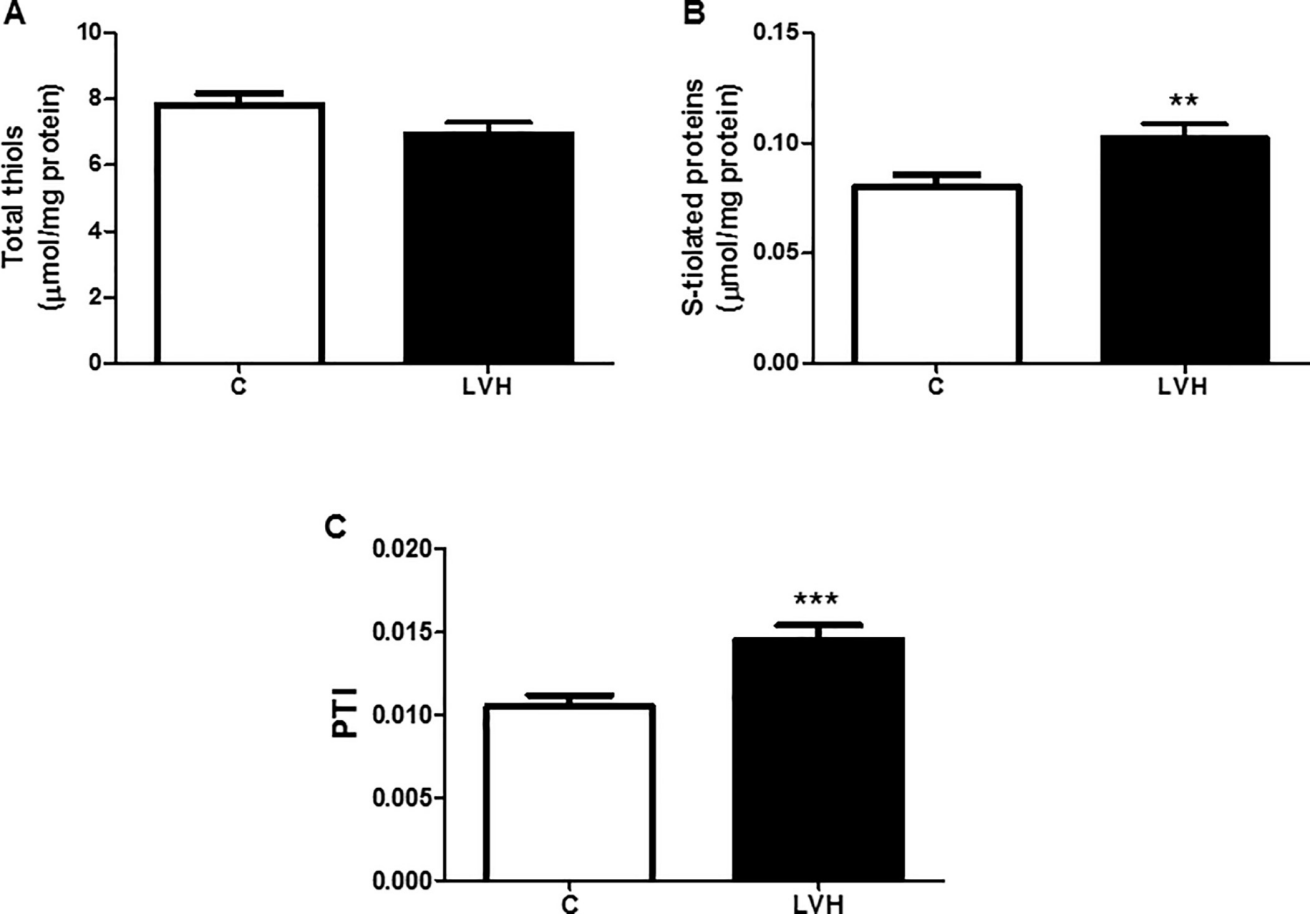
Protein thiolation index in patients with left ventricular hypertrophy. Association plasma total thiols (A), S-thiolated proteins (B), and Protein thiolation index (PTI) (C) to the presence of left ventricular hypertrophy; data are expressed as the mean ± SD; statistically significant differences between control group (C) and left ventricular hypertrophy group (LVH) are shown (^***^P < 0.001).

The oxido-reductive status of the subjects under study is shown in Fig 1. The new method for detecting plasma S-thiolated proteins showed an increase in LVH with respect to the control group (0.102±0.035 vs. 0.07±0.035 μmol/mg protein, P = 0.01). There were no differences in total thiols between the LVH group and the control group (7.414±2.250 and 7.819±2.053 μmol/mg protein, respectively). Finally, PTI was increased in patients with LVH with respect to the control group (0.014±0.005 vs. 0.010±0.004, P = 0.001).

The coefficient of variation was 40.9% and the minimun detection limit was 0.001771.

**Fig 2:**
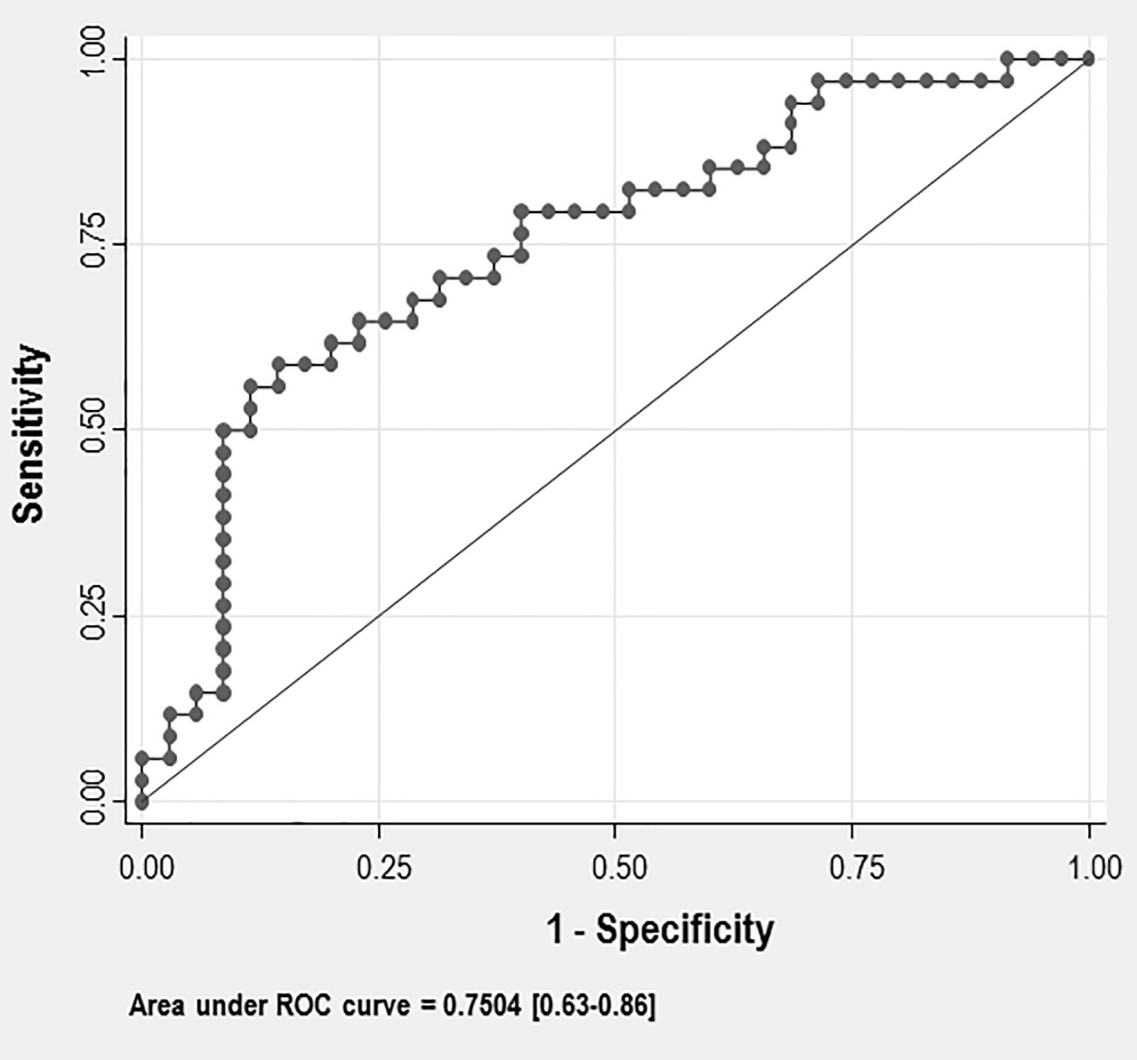
Receiver operating characteristic curve. Receiver operator curve (ROC) analysis for protein thiolation index (PTI) to diagnose left ventricular hypertrophy.

The area under the ROC curve (0.75, 95% confidence interval [CI] 0.63–0.86; P<0.001) shows the usefulness of the plasma PTI concentration in the diagnosis of LVH (Fig 2). At a cut-off value of 0.012, the 70.6% sensitivity and 68.6% specificity suggested that PTI could be used to screen for LVH. As this is an observational study, we show a positive predictive value of 68.6% and a negative predictive value of 70.6% for this cut-off (Table 2). We used the sensitivity and specificity of the cut-off to calculate the positive likelihood ratio (2.25) and negative likelihood ratio (0.43) (Table 2).

**Table 2:** Indexes of diagnostic validity of protein thiolation index for left ventricular hypertrophy.

PTI Cut-off	Sensitivity % (95% CI)	Specificity % (95% CI)	PPV % (95% CI)	NPV % (95% CI)	LR+ (95% CI)	LR- (95% CI)
0.012	70.6 (52.5–84.9)	68.6 (50.7–83.1)	68.6 (50.7–83.1)	70.6 (52.5–84.9)	2.25 (1.32–3.84)	0.43 (0.24–0.76)

PTI, protein thiolation index; PPV, positive predictive value; NPV, negative predictive value; LR+, positive likelihood ratio; LR-, negative likelihood ratio.

In order to study the association between PTI and LVH, we performed a multivariable logistic regression analysis adjusted for gender, age, smoking, diabetes mellitus, hypertension, dyslipidemia, renal failure, obesity, coronary disease, and valvular disease. As shown in Table 3, a statistically significant positive association (P = 0.02) was observed between PTI and LVH, independently of the variables described in the model.

**Table 3:** Multivariable logistic regression model: association protein thiolation index and left ventricular hypertrophy.

	OR (95% CI)	*P*
PTI	1.243 (1.031–1.497)	0.022
Gender	3.394 (0.609–18.917)	0.163
Age	1.032 (0.962–1.108)	0.379
Smokers	5.156 (0.517–51.443)	0.162
Glucose	0.994 (0.971–1.017)	0.614
Systolic arterial pressure	1.102 (1.030–1.179)	0.005
Diastolic arterial pressure	0.944 (0.872–1.022)	0.157
Dyslipidemia	1.467 (0.252–8.557)	0.670
Estimated glomerular filtration rate	0.988 (0.961–1.016)	0.410
Body mass index	1.038 (0.909–1.186)	0.580
Valvular and/or coronary disease	5.276 (1.023–27.211)	0.047

PTI (x1000), protein thiolation index

## Discussion

### PTI and LVH in humans

The main findings of our study were that patients with LVH had a higher PTI value than patients without LVH and that PTI level was independently associated with risk factors (gender, age, smoking, diabetes mellitus, hypertension, dyslipidemia, renal failure, obesity, coronary disease, and valvular disease). Therefore, this is the first study to propose PTI as a new biomarker of oxidative stress in patients with LVH.

Previous studies have revealed that LVH is one of the most important risk factors for cardiovascular disease (LVH is associated with a risk of cardiovascular events 2 to 4 times greater than in the healthy population) [8,26–28]. Therefore, diagnosis of LVH is important if we are to minimize the impact of cardiovascular disease. Methods for diagnosing LVH include ECG, echocardiography, and cardiac magnetic resonance [29]. ECG is an accessible and inexpensive method for the diagnosis of LVH, however the sensitivity of ECG ranges from 7% to 35% for mild LVH to 30% to 60% for moderate to severe LVH [30]. Although not immune from technical limitations, echocardiography is more sensitive than ECG in diagnosing LVH and has been regarded as a diagnostic gold standard [24]. Cardiac magnetic resonance is considered more accurate for estimated left ventricular mass compared to echocardiography, however it is less commonly used because it has higher cost and is less feasible and available [31]. Thus, the low sensitivity, lengthy diagnostic, poor cost-effectiveness ratio, and technical complexity highlight the need for new methods to diagnose LVH. As an alternative, biomarkers could play a relevant role in diagnosis of LVH. Biomarkers are an indicator of pathogenic processes. They indicate the presence/absence of disease and can be used to monitor disease development and progression and response to treatment [32]. Several studies have evaluated natriuretic peptides for the diagnosis of LVH, but the results are contradictory [33–37]. This might be due to the heterogeneity of the populations included, the type of natriuretic peptide analized, and the method used to confirm LVH. Analysis of the usefulness of plasma NT-proBNP (N-terminal pro-brain natriuretic peptide) concentrations in the diagnosis of LVH shows an area under the ROC curve of 0.75 with 76.6% sensitivity and 65.7% specificity [33]. However, only patients who presented hypertension without systolic dysfunction were included. A direct correlation was found between cardiotrophin-1 and left ventricular mass index, with an area under the ROC curve of 0.78, with 70% sensitivity and 75% specificity for predinting LVH [38]. Serum miR-27b which was significantly elevated in hypertensive patients with LVH from hypertensive patients without LVH, could be used to screen for LVH (AUC of 0.81 with 79.1% sensitivity and 70.3% specificity) [29]. Oxidative stress has an important pathophysiological role in patients with LVH [19]. Clinical studies have determinated correlations between biomarkers of oxidative stress and LVH: Galectin-3 has increased in patients with LVH related to hypertension [39] and serum oxidized low-density lipoproptein shows correlation with LVH related to chronic kidney disease [40]. However, these biomarkers are not commonly used in the LVH diagnosis, thus the role of oxidative stress in patients with LVH is underestimated in clinical practice. Consequently, biomarkers with inexpensive reagents that can be implemented easily and quickly and are easily measurable (plasma biomarkers) are needed to facilitate routine measurements of oxidative stress in patients with LVH. Unfortunately, it is difficult to find biomarkers with such characteristics [41–42]. PTI is a new biomarker of oxidative stress [18,43]. However, no association has been established with LVH. In the present study, we used a new spectrophotometry method, which has been validated by other authors, to analyze PTI [18]. The technique is easy to perform and suitable for clinical analyses with a high daily throughput, thus enabling rapid assessment.

Oxidative stress is a complex problem that is the consequence of an imbalance between oxidative damage and antioxidant defense, where many biomarkers play a role [44]. In addition, the complex and multifactorial nature of oxidative stress makes it difficult to assign a prevalent role to a particular biomarker in a disease and does not provide information on overall oxidative status (oxidant and antioxidant) [45]. However, given that PTI is defined as the molar ratio of S-thiolated proteins (oxidant agents) to free protein thiols in plasma (antioxidant molecules) [18], it includes biomarkers of oxidative damage and antioxidant defense.

### Strengths and limitations

Our study is subject to a series of limitations. First, we examined the association between determination of plasma PTI and LVH in order to monitor the development of LVH. We intend to use this approach to check the efficiency of treatment in patients with heart disease (coronary and/or valvular). Therefore, it would be interesting to reproduce the results obtained in this study in patients with hypertension only (since this is the most frequent cause of LVH) in order to be able to use PTI for screening of LVH in the general population. Second, we evaluated LVH using echocardiography. The interobserver variability of this technique could lead to bias [46] thus, echocardiography should be performed by single cardiologist, however echocardiography was performed by a small group of experienced cardiologists. Finally, the premise of our investigation is that screening with PTI could be less expensive than standard echocardiography; however, the high quality of echocardiography may reduce the cost gap between both approaches [47].

## Conclusion

Our findings suggest that PTI could be a new biomarker of oxidative stress in patients with LVH. It remains necessary to validate the clinical utility of PTI using a large, blinded set of samples and to establish comparability and standards for quality control in order to monitor the development of LVH and to check the efficiency of treatment in the general population.
